# Mitral valve repair using the free-knot technique: A simple, precise, and length-stable method for expanded polytetrafluoroethylene chordal adjustment

**DOI:** 10.1016/j.xjtc.2026.102409

**Published:** 2026-05-05

**Authors:** Yuki Wada, Takehiko Matsuo, Akira Marui, Shinichi Tsumaru, Keisuke Hakamada, Yuki Kuroda, Yuta Kitagata, Ryo Imada, Nobuhisa Ohno

**Affiliations:** Department of Cardiovascular Surgery, Kokura Memorial Hospital, Kitakyusyu, Japan

**Keywords:** mitral valve repair, mitral valve regurgitation, artificial chordae implantation

## Abstract

**Objectives:**

Artificial chordal implantation using expanded polytetrafluoroethylene (ePTFE) is widely adopted in mitral valve repair (MVr). However, achieving precise chordal length remains challenging because durable valve competence depends on fine intraoperative adjustment. Moreover, ePTFE sutures are slippery and may change in length during knot-tying, making stable fixation difficult. The free-knot technique enables incremental intraoperative length adjustment and maintains the intended length without alteration during final knot fixation. This study aimed to describe the free-knot technique and evaluate its short-term clinical and echocardiographic outcomes.

**Methods:**

We retrospectively analyzed 118 consecutive patients who underwent MVr using the free-knot technique between April 2021 and March 2025 at a single institution.

**Results:**

The mean age was 59.4 ± 11.4 years, and 48% were male. Posterior, anterior, and bileaflet prolapses were present in 66.1%, 17.8%, and 16.1% of patients, respectively. All patients underwent annuloplasty, with a mean of 2.3 ± 1.1 artificial chordae implanted. None of the patients had moderate or severe residual mitral regurgitation findings at discharge. Freedom from recurrent moderate or greater mitral regurgitation was 92.9% at 1 year, 90.2% at 2 years, and 85.2% at 3 years. Freedom from severe MR and reoperation remained 100% throughout follow-up, with no significant difference in MR recurrence according to the prolapse site.

**Conclusions:**

The free-knot technique is a simple, adjustable, and device-free method that ensures accurate chordal length tuning and stable fixation without length changes during knot-tying. This approach achieved excellent short-term outcomes across diverse prolapse sites, supporting its value as a versatile tool for contemporary MVr.

**Figure:**
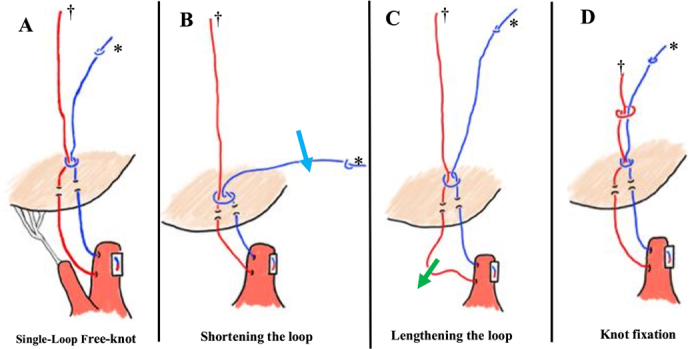
Precise ePTFE chordal length adjustment and stable fixation using the Free-knot technique.

**Central Message**

The free-knot technique enables device-free adjustment and knot tying of ePTFE artificial chordae, maintaining length stability and achieving durable mitral valve repair across diverse prolapse sites.

**Perspective**

The free-knot technique addresses 2 key challenges in artificial chordal repair: precise intraoperative length adjustment and length stability during knot tying—without dedicated devices. By simplifying these steps, it enhances the reproducibility and reliability of mitral valve repair using ePTFE artificial chordae.

Mitral valve repair (MVr) is the standard treatment for patients with mitral regurgitation (MR) caused by leaflet prolapse secondary to degenerative disease, because numerous studies have demonstrated superior survival and clinical outcomes with MVr compared with mitral valve replacement.^1^, ^2^, ^3^, ^4^ Two major techniques are widely accepted for the repair of degenerative MR: the resection and suture technique and the artificial chordae implantation technique.^5^^,^^6^ Artificial chordae implantation using expanded polytetrafluoroethylene (ePTFE) is an important and established technique for MVr, and this technique shows excellent mid- and long-term outcomes in several reports.^6^, ^7^, ^8^ ePTFE sutures are known to be slippery, and their smooth surface can cause subtle slippage or length alteration during knot-tying, potentially affecting the precision of chordal adjustment. To address these issues, various technical modifications for artificial chordal replacement using ePTFE sutures have been reported, including different methods of suture placement on the papillary muscle and leaflet, knot fixation, and the use of dedicated devices.^6^^,^^9^, ^10^, ^11^, ^12^ These efforts have led to the development of techniques that allow fine intraoperative length control and secure fixation without tension-related changes. Therefore, this study aimed to present artificial chordae implantation using the free-knot technique with ePTFE chordae for the correction of mitral valve prolapse and to report its short-term outcomes.

## Methods

### Ethics Statement

This study was conducted in accordance with the principles of the Declaration of Helsinki and approved by the Ethics Committee of Kokura Memorial Hospital (ID: 25072201; date: July 22, 2025). Written informed consent was obtained from all patients for the publication of this report.

### Patient Population

We retrospectively reviewed the data of 118 patients who underwent MVr using artificial chordae implantation with the free-knot technique at our institution between April 2021 and March 2025. Patients were included regardless of whether they underwent concurrent procedures, including coronary artery bypass grafting, other valvular surgeries, arrhythmia correction procedures, or congenital heart disease repair.

### Surgical Procedure

Surgeries were performed via either median sternotomy or right minithoracotomy (minimally invasive surgery or robotic surgery). In the median sternotomy group, arterial inflow was established through the ascending aorta and venous drainage via bicaval (superior vena cava/inferior vena cava) cannulation, whereas in the right minithoracotomy group, arterial perfusion was achieved via the femoral artery and venous drainage via the right internal jugular and femoral veins. In all cases, cardiac arrest was achieved using blood cardioplegia. The mitral valve was accessed via a right-sided left atriotomy or the superior transseptal approach.

**Figure 1:**
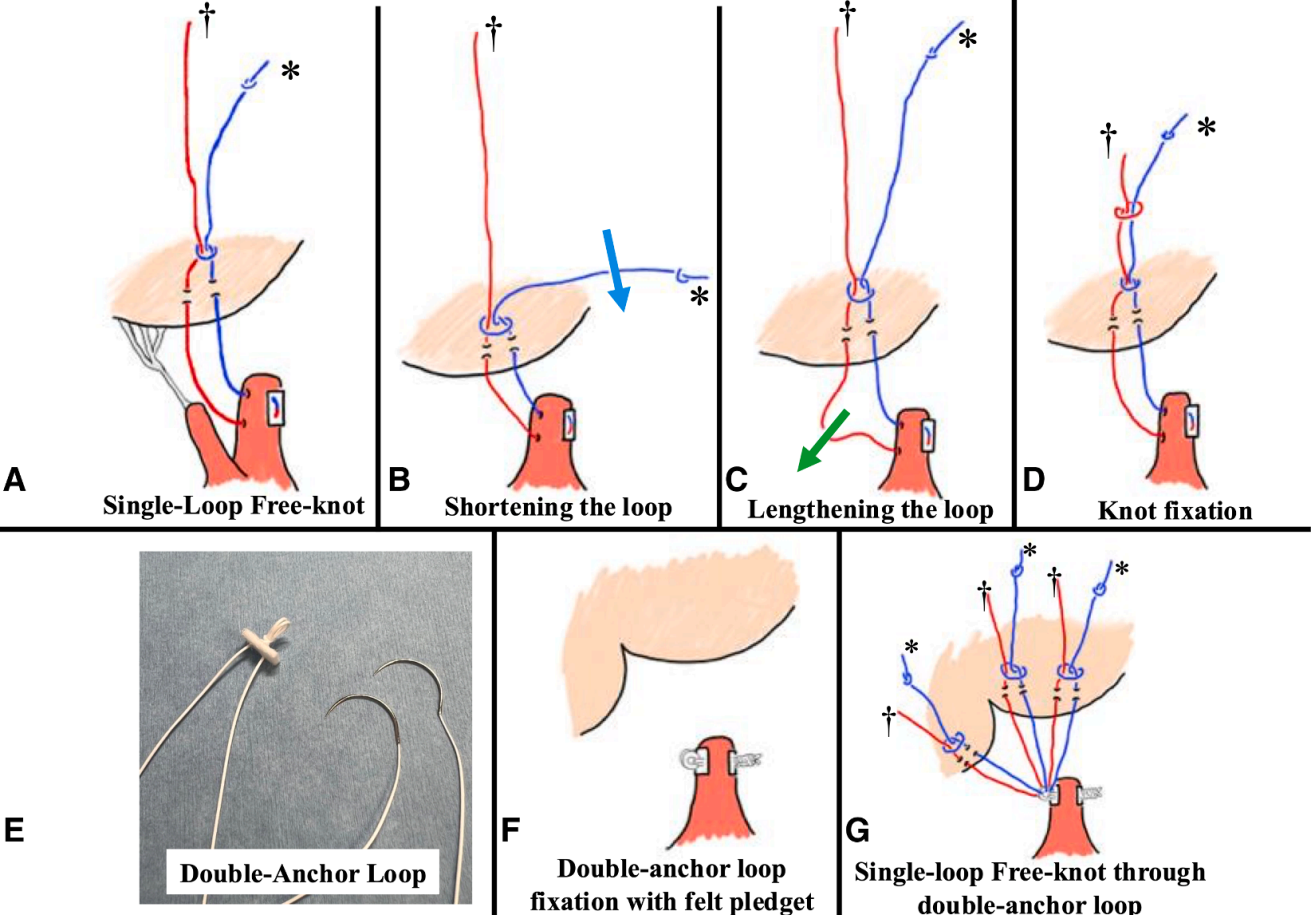
A, A pledgeted CV-4 expanded polytetrafluoroethylene suture was passed twice through the papillary muscle (tip and midbody) and placed through the prolapsing leaflet in a horizontal mattress fashion. The leaflet was secured with a single free-knot, and the primary strand (*blue*, ∗) was marked with a knot for subsequent length adjustments. B, Leaflet coaptation was assessed using a saline test after mitral annuloplasty. If an artificial chordae was too long, the secondary strand (*red*, †) was held fixed, and the primary strand (*blue* ∗) was tensioned to shorten the loop (*blue arrow*). C, Leaflet coaptation was assessed using a saline test after mitral annuloplasty. If too short, the primary strand (*blue*, ∗) was held fixed, and the secondary strand (*red*, †) was released to lengthen the loop (*green arrow*). D, Once the optimal length was confirmed, the secondary strand (*red*, †) was tied down using the primary strand (*blue*, ∗) as a static anchor (this technique ensures that the adjusted length is not altered during knotting). E, A double-anchored loop was created using a CV-3 expanded polytetrafluoroethylene suture. F, A double-anchor loop was passed through the papillary muscle and secured with a felt pledget. G, Up to 5 CV-4 loops could pass through this anchor loop. Each loop was subsequently passed through the prolapsed leaflet and secured using the free-knot method, and its primary strand (*red*, ∗) was marked with a knot.

The extent of leaflet prolapse and condition of the subvalvular apparatus were carefully assessed, with particular attention paid to identifying the papillary muscle (anterolateral or posteromedial) corresponding to the ruptured or elongated chordae. Artificial chordal length was determined using the length of the adjacent intact native chordae as a reference, with final fine adjustment performed by repeated saline testing. Artificial chordae implantation was performed using the free-knot technique as follows (Video 1):

1. Single-loop free-knot (Figure 1, *A*).

Two needles of a pledgeted CV-4 ePTFE suture (W. L. Gore & Associates, Inc) were passed longitudinally through the papillary muscle: one through the fibrous tip and the other through the central aspect of the muscle body. Care was taken to avoid compromising papillary muscle perfusion, and the suture was left untied on the muscle. The needles were then passed through the prolapsing leaflet in a horizontal mattress manner and secured with a single free-knot tie. A small marker knot was placed on the primary strand (blue ∗) for identification during the final fixation.

2. Chordal length adjustment and knot fixation (Figure 1, *B-D*).

After mitral annuloplasty, leaflet coaptation was assessed by a saline test.

• If an artificial chordae was too long, the secondary strand (red, †) was held fixed, and the primary strand (blue, ∗) was tensioned to shorten the loop (Figure 1, *B*).
• If too short, the primary strand (blue, ∗) was held fixed, and the secondary strand (red, †) was released to lengthen the loop (Figure 1, *C*).

Once the optimal length was confirmed, the secondary strand (red, †) was tied down using the primary strand (blue, ∗) as a static anchor (this technique ensures that the adjusted length is not altered during knotting) (Figure 1, *D*). Before and after final knot tying, we routinely confirmed that the lengths of both ePTFE strands were appropriately aligned and that the adjusted chordal length was maintained.

3 .Double-anchor loop (for ≥3 artificial chordae implantation) (Figure 1, *E-G*).

When 3 or more artificial chordae implantations were required, a double-anchor loop was created using CV-3 ePTFE sutures (Figure 1, *E*). The loop passed through the papillary muscle and was secured using a felt pledget (Figure 1, *F*). Up to 5 CV-4 loops could pass through this anchor loop. Each loop was subsequently passed through the prolapsed leaflet and secured using the free-knot method, and its primary strand (blue, ∗) was marked with a knot (Figure 1, *G*).

4. Final testing and confirmation.

After the final saline and ink tests revealed satisfactory results, transesophageal echocardiography was routinely conducted immediately after weaning from cardiopulmonary bypass to confirm the adequacy of MVr. All patients underwent transthoracic echocardiography before discharge and at 1 year postoperatively, with annual follow-ups as feasible. Postoperative follow-up was primarily based on transthoracic echocardiography. Transesophageal echocardiography was performed selectively when further evaluation was required, whereas magnetic resonance imaging was not used in this series.

### Definitions

#### Study end points

The primary end point was a composite of recurrent MR greater than moderate severity or the need for mitral valve reoperation during the follow-up period. The secondary outcomes included all-cause mortality and cardiac death.

### Statistical Analysis

All statistical analyses were performed using R software (version 4.3.1; R Foundation for Statistical Computing). Continuous variables are presented as mean ± standard deviation or as median with interquartile range. Group comparisons of continuous variables were performed using the Mann-Whitney *U* test, whereas categorical variables were compared using the Fisher exact test. Kaplan-Meier survival analysis and log-rank tests were used to determine the survival and event-free rates. The data underlying this article will be shared upon reasonable request from the corresponding authors.

## Results

### Patient Characteristics

A total of 118 patients were included in the study, with a mean age of 59.4 ± 11.4 years and a mean body surface area of 1.65 ± 0.20 m^2^. Forty-eight percent were male, and the common comorbidities included hypertension (48.3%), dyslipidemia (29.7%), chronic kidney disease (23.7%), and diabetes mellitus (5.9%) (Table 1).

**Table 1:** Patient characteristics

Variables	n = 118
Age, y	59.4 ± 11.4
Male	67 (48.5)
BSA, m^2^	1.65 ± 0.20
Hypertension	57 (48.3)
Diabetes mellitus	7 (5.9)
Chronic kidney disease (Cre >1.3 mg/dL)	28 (23.7)
Hemodialysis	0 (0)
Dyslipidemia	35 (29.7)
Chronic obstructive pulmonary disease (>moderate)	6 (5.1)
Chronic or paroxysmal atrial fibrillation	12 (21.1)
Emergent/urgent surgery	0 (0)
Redo	0 (0)
Mitral valve pathology	
Degenerative	113 (95.8)
Healed infective endocarditis	5 (4.2)
Active infective endocarditis	0 (0)
Rheumatic	0 (0)
Congenital	0 (0)
Preoperative echocardiographic findings	
Left ventricular diastolic dimension, mm	54.1 ± 5.6
Left ventricular systolic dimension, mm	35.0 ± 4.7
Ejection fraction, %	64.1 ± 5.5
Prolapse leaflet	
Posterior leaflet	78 (66.1)
Anterior leaflet	21 (17.8)
Bileaflet	19 (16.1)

*BSA*, Body surface area; *Cre*, creatine.

#### Mitral valve pathology

Most patients (95.8%) had degenerative disease, and 4.2% had healed infective endocarditis. No active endocarditis, rheumatic, or congenital disease was observed.

#### Preoperative echocardiographic findings

The mean left ventricular end-diastolic dimension was 54.1 ± 5.6 mm, and the mean end-systolic dimension was 35.0 ± 4.7 mm. The mean left ventricular ejection fraction was well preserved at 64.1 ± 5.5%.

#### Prolapsed leaflet

Posterior leaflet prolapse was the most frequent lesion, affecting 78 patients (66.1%). Anterior leaflet prolapse was present in 21 patients (17.8%), whereas bileaflet prolapse was observed in 19 patients (16.1%).

### Operative and Procedure Details

All patients underwent MVr with concomitant annuloplasty (100%) (Table 2). Median sternotomy was performed in 18 patients (15.3%), minimally invasive surgery in 57 patients (48.3%), and robot-assisted surgery in 43 patients (36.4%). A right-sided left atriotomy was the standard approach to the left atrium (98.3%), whereas a superior transseptal incision was used in 2 patients (1.7%). The mean number of artificial chordae implanted was 2.3 ± 1.1, with a mean of 2.0 (range, 1-4) for posterior leaflet repair, 2.1 (range, 1-4) for anterior leaflet repair, and 3.1 (range, 1-8) for bileaflet prolapse.

**Table 2:** Operative and technical data

Variables	n = 118
Approach	
Median sternotomy	18 (15.3)
MICS	57 (48.3)
Robotic	43 (36.4)
Approach to the left atrium	
Right-sided left atriotomy	116 (98.3)
Superior transseptal	2 (1.7)
No. of artificial chordae	2.3 ± 1.1
Posterior leaflet	2.0 (1-4)
Anterior leaflet	2.1 (1-4)
Bileaflet	3.1 (1-8)
Mitral annuloplasty	118 (100)
Additional techniques for MVr	
Indentation closure	19 (16.1)
Commissural edge-to-edge	28 (23.7)
Resection and suture	25 (21.2)
Folding plasty	3 (2.5)
Patch augmentation	1 (0.8)
Concomitant procedure	
Aortic valve	3 (2.6)
Tricuspid valve	37 (36.3)
CABG	2 (1.7)
Arrhythmia	20 (16.9)
Aorta	1 (0.8)

*MICS*, Minimally invasive cardiac surgery; *MVr*, mitral valve repair; *CABG*, coronary artery bypass grafting.

Additional techniques were applied in selected cases, including commissural edge-to-edge repair in 28 patients (23.7%), resection and suturing in 25 (21.2%), indentation closure in 19 (16.1%), folding plasty in 3 (2.5%), and patch augmentation in 1 (0.8%). Concomitant procedures were performed in 61 (51.7%) patients. The most common surgeries were tricuspid valve repair in 37 patients (36.3%) and arrhythmia surgery in 20 patients (16.9%). Other concomitant procedures included aortic valve replacement in 3 patients (2.6%), coronary artery bypass grafting in 2 (1.7%), and aortic replacement in 1 (0.8%).

### Residual and Recurrent MR

**Figure 2:**
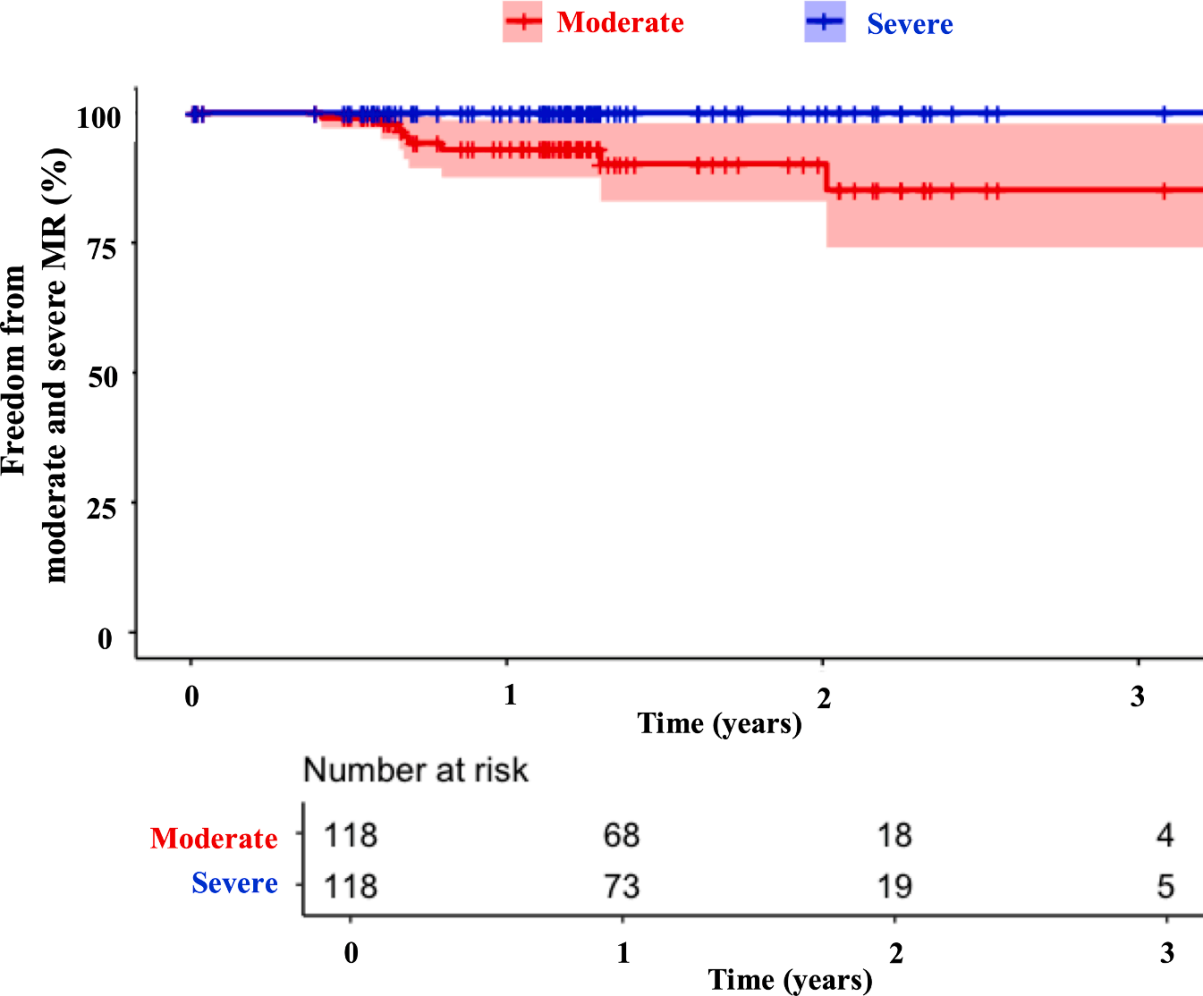
Freedom from moderate and severe MR assessed by echocardiography. *Shaded areas* indicate 95% CIs. *MR*, Mitral regurgitation.

**Figure 3:**
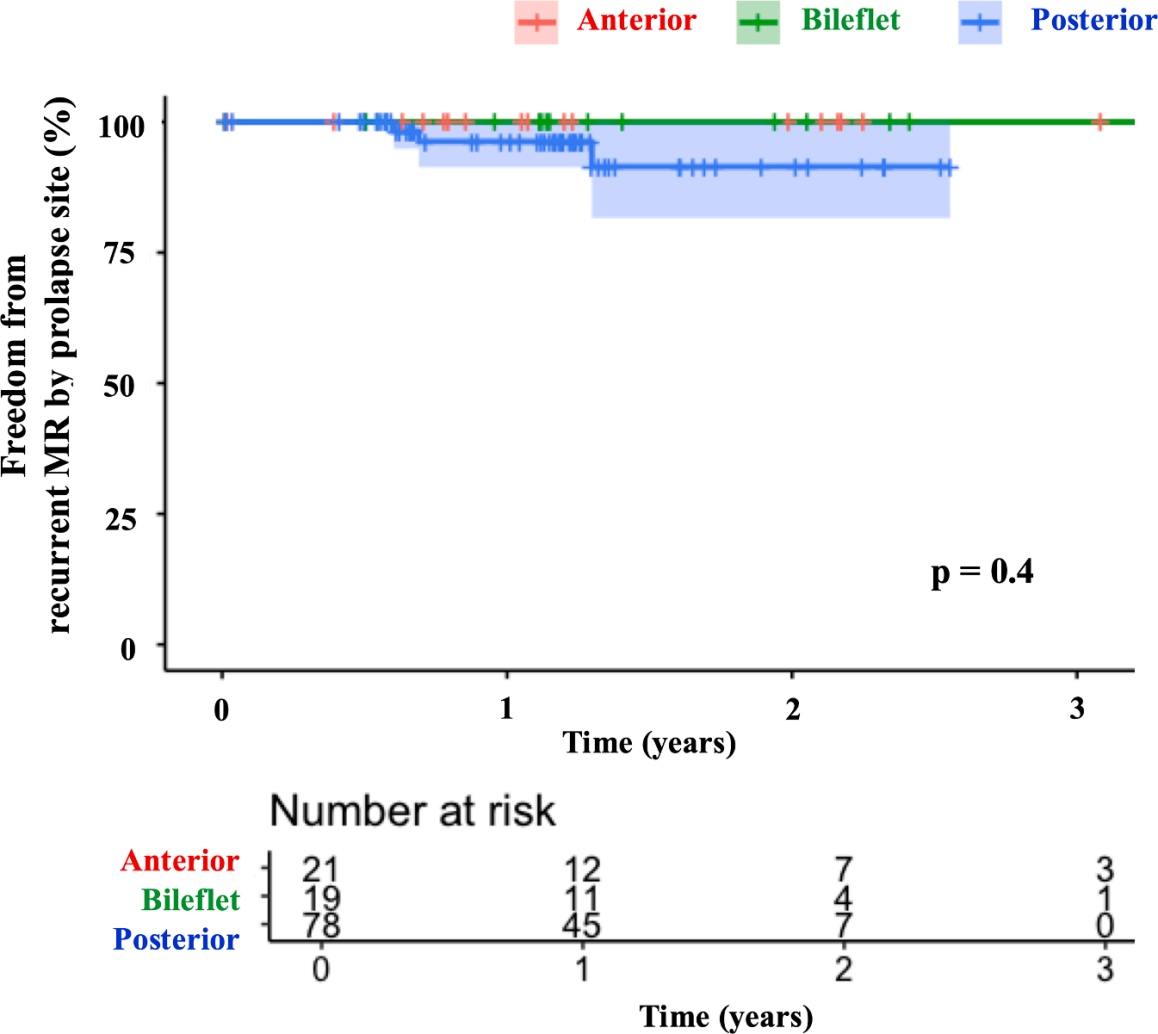
Freedom from recurrent MR, as defined in the study end point (greater than moderate or requiring reoperation), according to the site of the prolapsed leaflet. *Shaded areas* indicate 95% CIs. *MR*, Mitral regurgitation.

At discharge, MR was none or trivial in 82 patients (69.5%), mild in 34 patients (28.8%), and mild-to-moderate in 2 patients (1.7%) (Table 3). No patient had moderate-to-severe residual regurgitation. During follow-up, freedom from moderate and severe MR as assessed by echocardiography is shown in Figure 2. Freedom from moderate MR was 92.9% at 1 year, 90.2% at 2 years, and 85.2% at 3 years postoperatively, respectively. The rate of freedom from severe MR was 100% throughout the follow-up period. None of the patients required reoperation during the follow-up period. Freedom from recurrent MR, as defined at the study end point (greater than moderate or requiring reoperation) according to the site of the prolapsed leaflet, is shown in Figure 3. No significant differences in MR recurrence were observed between the anterior, posterior, and bileaflet prolapse groups. Among the patients who developed recurrent MR of at least moderate severity, 4 underwent further evaluation with transesophageal echocardiography. In these cases, no recurrent prolapse was identified at the repair site, and the recurrent MR appeared to be related to lesions at other sites.

**Table 3:** Clinical findings at discharge

Variables	n = 118
Hospital stay, d	16.1 ± 6.6
ICU stay, d	1.3 ± 0.57
Hospital death	0 (0)
MR at discharge	
None/trivial	82 (69.5)
Mild	34 (28.8)
Mild to moderate	2 (1.7)
Moderate	0 (0)
Severe	0 (0)

*ICU*, Intensive care unit; *MR*, mitral regurgitation.

**Figure 4:**
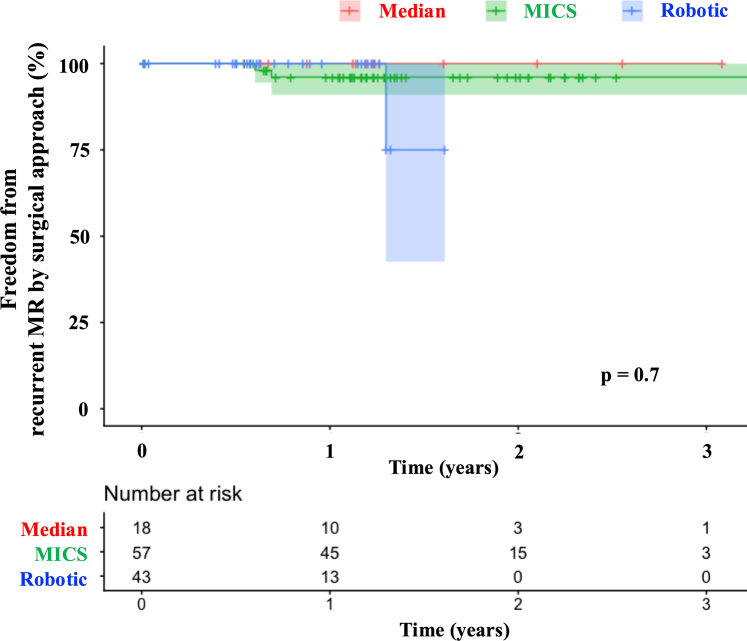
Freedom from recurrent MR, defined as greater than moderate MR or mitral valve reoperation, according to surgical approach. *Shaded areas* indicate 95% CIs. *MR*, Mitral regurgitation; *MICS*, minimally invasive cardiac surgery.

Freedom from recurrent MR according to surgical approach is shown in Figure 4. No significant differences in MR recurrence were observed among the median sternotomy, minimally invasive cardiac surgery, and robotic surgery groups.

### Mortality

No in-hospital deaths occurred. The mean intensive care unit stay was 1.3 ± 0.6 days, and the mean postoperative hospital stay was 16.1 ± 6.6 days. During the follow-up period, no deaths occurred, including cardiac deaths. In addition to echocardiographic follow-up, postoperative clinical events after discharge were reviewed. One patient was readmitted to the hospital for heart failure attributable to pericardial effusion at 0.15 years after surgery. Another patient who had undergone concomitant arrhythmia correction surgery developed recurrent atrial fibrillation 2 years after surgery and underwent catheter ablation. No other cardiac-related events were identified during follow-up.

## Discussion

### Principal Findings

This study demonstrated that MVr using the free-knot technique with ePTFE artificial chordae implantation achieved excellent short-term outcomes, with no in-hospital mortality, no reoperation, and 100% freedom from severe MR throughout the follow-up. Freedom from recurrent MR greater than moderate remained at 85.2% at 3 years, which is comparable with the outcomes reported for conventional ePTFE artificial chordae implantation in previous studies.^6^, ^7^, ^8^ Furthermore, no significant difference in MR recurrence was observed across anterior, posterior, and bileaflet prolapses, suggesting that this method provides consistent and durable repair even in anatomically demanding lesions.

### Technical Considerations

Accurate adjustment of the artificial chordal length is essential for achieving durable mitral valve competence, as even subtle discrepancies may lead to residual or recurrent MR. However, ePTFE sutures have a smooth and slippery surface, making knot security and precise length control technically challenging. To address these challenges, a wide spectrum of methods for determining or stabilizing the chordal length has been proposed. Early efforts focused on reproducing native chordal geometry, including the premeasured Gore-Tex loop system introduced by von Oppell and Mohr.^13^ Other authors emphasized intraoperative measurement tools, such as intracardiac calipers or the slit-stent technique.^14^^,^^15^ Calafiore^16^ proposed a leaflet-traction method in which the anterior leaflet is pulled up to its maximum height with nerve hooks, and the artificial chordae are tied with an additional 5 mm added to the free margin. This concept highlights the importance of maintaining physiological leaflet–annulus relationships rather than relying solely on absolute chordal measurements.

Techniques aimed at fine intraoperative tuning, such as the length-adjustment method described by Maselli and colleagues,^17^ further underscore the need for precision in chordal reconstruction. Simplified implantation strategies, including clip-and-tie fixation and direct reconstruction approaches, have also been reported.^18^^,^^19^ Together, these methods reflect ongoing efforts to improve reproducibility and durability in artificial chordal repair. In contrast to approaches requiring additional devices or predetermined lengths, the free-knot technique enables stepwise intraoperative fine adjustment and secure fixation without tension-related length changes, using a simple, device-free method applicable to all surgical approaches. The comparable outcomes across anterior, posterior, and bileaflet lesions in the present study suggest that stable and precise chordal adjustment can be reliably achieved using this technique.

Although pledget reinforcement may theoretically affect immediate strand equalization, pledgeted sutures were used because they were considered more protective for the papillary muscle. Given the smooth surface characteristics of ePTFE, the impact of pledget-related resistance is likely to be limited, although this possibility cannot be completely excluded. In addition, although longitudinal passage of the suture through the papillary muscle may theoretically raise concern for subsequent suture migration and chordal lengthening, the distance between the 2 penetration points is very short in our technique, and the impact of this configuration is also likely to be limited.

### Comparison With Previous Reports

The long-term durability of ePTFE chordal replacement is well established, with David and colleagues^6^ and Hata and colleagues^7^ reporting excellent survival and freedom from reoperation over decades. Large series, such as the 608-patient cohort by Salvador and colleagues,^8^ further support the reliability of ePTFE chordae for degenerative MR.

Our short-term outcomes align with these previous findings and suggest that the free-knot technique achieves similar valve competence without adding technical complexity. Compared with loop-based or adjustable systems, the free-knot technique integrates the advantages of fine-tuning and secure fixation into one simplified step. This may reduce operative variability among surgeons and facilitate the broader adoption of artificial chordal repair, particularly in minimally invasive or robotic approaches, where instrumentation constraints amplify the importance of reliable knot stability.

### Clinical Implications

The versatility of the free-knot technique, which is applicable via sternotomy, mini-thoracotomy, or robotic surgery, strengthens its clinical applicability. Precise chordal adjustment under saline testing is intuitively performed, and the method does not rely on proprietary devices or additional materials, which may be advantageous in terms of cost-effectiveness and reproducibility. Our findings reinforce the idea that durable MR elimination can be achieved using a technique that remains simple, easily teachable, and adaptable to complex leaflet pathologies.

### Limitations and Future Directions

This study had some limitations. This was a retrospective, single-center study with a modest sample size and a relatively short follow-up duration. In addition, the exact mechanism of recurrent MR was difficult to determine in this retrospective series. In most cases, transthoracic echocardiography alone was insufficient for detailed mechanistic assessment, and no patient underwent reoperation for direct confirmation. The echocardiographic evaluation intervals were not entirely standardized, and subclinical MR progression may have been underestimated. The long-term durability beyond 3 years remains to be determined. Future multicenter studies with larger cohorts and extended follow-ups are required to validate these findings and further assess the long-term performance and generalizability of the free-knot technique.

## Conclusions

In summary, the free-knot technique is a simple, reproducible, and adjustable method for artificial chordal implantation of ePTFE, yielding excellent short-term outcomes and reliable valve competence across a broad range of prolapse lesions. This technique may be a valuable addition to contemporary mitral valve repair strategies.

## Conflict of Interest Statement

The authors reported no conflicts of interest.

The *Journal* policy requires editors and reviewers to disclose conflicts of interest and to decline handling or reviewing manuscripts for which they may have a conflict of interest. The editors and reviewers of this article have no conflicts of interest.

## Supplementary Data

Video 1 Free-knot technique for artificial chordal implantation. The video demonstrates the following steps: (1) Single-loop free-knot. (2) Chordal length adjustment, including shortening and lengthening of the loop, followed by final knot fixation. (3) Double-anchor loop technique using four single-loop free-knots. (4) Final saline testing. Video available at: https://www.jtcvs.org/article/S2666-2507(26)00216-6/fulltext.
